# Climate change impacts on livestock in Brazil

**DOI:** 10.1007/s00484-024-02778-3

**Published:** 2024-09-23

**Authors:** Ferreira N. C. R., Andrade R. R., Ferreira L. N.

**Affiliations:** 1 Natural Hazards & Environment R&D, EDF Energy, London, W1T 4EZ UK; 2 Department of Biosystems Engineering, College of Agronomy, Federal University of Goiás, 74690- 900 Goiânia, GO Brazil; 3 Big Data Institute, University of Oxford, Oxford, OX3 7LF UK

**Keywords:** Animal heat stress, Climate change, Food security, Livestock, Temperature-humidity index

## Abstract

Brazilian livestock provides a significant fraction of the food consumed globally, making the country one of the largest producers and exporters of meat, milk and eggs. However, current advances in the production of protein from Brazilian animal origin may be directly impacted by climate change and the resulting biophysical effects. Therefore, it is strategically consistent to develop measures to deal with the resulting environmental heat stress on domesticated animal species, especially the need in developing countries. This work aims to (1) evaluate the impacts of climate change on livestock (cattle-dairy, cattle-beef, goats, sheep, pigs, poultry-general) in different regions of Brazil and (2) discuss possible response strategies, associated with animal comfort and welfare. From our results, we can draw better strategies to mitigate the impacts of climate change on livestock production. The results presented show an increase of high heat stress in South and Southeast and an increase of extreme heat stress in North and Central-West areas of Brazil. The rise in extreme heat stress tends to occur mostly during spring and summer and tends to vary considering the different evaluated species. Within the evaluated species, the ones that seem to be more affected by climate changes are *Poultry*, *pigs*, *cattle-beef* and *general* (temperature-humidity index value)*.* The differences between the results for the five geographic regions in Brazil suggests that different mitigation measures need to be considered to cope with future heat stress in livestock. To ensure the long-term success of Brazil's influence on the global market for proteins of animal origin, it must achieve sustainable production systems more intensively.

## Introduction

Climate change and extreme events affect different regions globally, with a range of negative impacts affecting multiple sectors, such as health (Vicedo-Cabrera et al. 2021), energy (Jong et al. 2019; Ferreira et al. 2023a), and agriculture (Zilli et al. 2020; Appiah et al. 2023; Ferreira et al. 2023b). As an impact of climate change, we expect an increase in temperature, changes in relative humidity and heatwaves. All of these changes will affect livestock differently across the world (Allen et al. 2015).The main consequence is a stronger heat stress (Vitali et al. 2015). Heat stress in domesticated livestock occurs when environmental conditions challenge the animal's thermoregulatory mechanisms (Thornton et al. 2021). The effects of heat stress include reduced production, reproduction, fertility, animal welfare, increased susceptibility to disease and, in some cases, increased mortality (Herbut et al. 2021; Andrade et al. 2022a). If our global society does not reduce protein of animal origin consumption in the near future, then the additional heat stress might also compromise food security in the coming years. Brazilian agricultural and stock breeding production provides a significant fraction of the food consumed worldwide (Zilli et al. 2020).

Climate models are important tools to assess climate change impacts. Projections from Phase Six of the Coupled Model Intercomparison Project (CMIP6) provide climate scenarios based on different Shared Socio-Economic Pathways (SSP), and are widely used to assess climate change impacts in different sectors (Thornton et al. 2021; Zeng et al. 2022). Using climate data, available as an output of climate models, heat stress indices can be calculated to investigate the impact of climate change on livestock, in different regions, and different species (Berman, 2019). The temperature-humidity index (THI) is one of the indexes that are frequently used to represent the heat stress for different species (Allen et al. 2015; Oliveira et al. 2022). Exploiting existing variation in heat tolerance among different animal species could be a key adaptation strategy (Thornton et al. 2021). Measures that assess animal welfare, environmental and economic issues are little investigated in the context of climate change (Hempel et al. 2019). At the same time, there are still rare studies in the literature that estimate the effect of climate change on Brazilian livestock, especially paying attention to the different regions of the country, taking into account the large territorial extension. Literature suggests an emphasis on mitigation strategies, including nutrition and genetics, as well as housing strategies and cooling mechanisms (e.g. ventilation and sprinkling) to reduce the effect of heat stress on animals (Garcia et al. 2015; Guesine et al. 2023).

The goal of this paper is to evaluate the impacts of climate change by using the THI and specific thresholds to different livestock species (cattle-dairy, cattle-beef, goats, sheep, pigs, poultry-general). This impact was evaluated by using CMIP6 ensemble models for historical period, and short-, medium- and long-term projections. For this analysis, we used the SSP5-8.5 scenario which assumes a business as usual scenario. We focused on the five geographical regions in Brazil, as different regions have different productions and will be affected differently by climate change.

## Material and methods

### Datasets: CMIP6 climate change projections

Projections from Phase Six of the Coupled Model Intercomparison Project (CMIP6) provide climate scenarios based on different Shared Socio-Economic Pathways (SSP) (O’Neill et al. 2014, 2020). The SSP scenarios were created from long-term integrations with possible greenhouse gas emission scenarios in the atmosphere and their impacts on climate variables. These scenarios can be used to investigate the implications of long term climatic changes for designing robust policies in an environment of interacting complex systems and uncertainty (Hall et al. 2016; Harrison et al. 2015; O’Neil et al. 2014). These scenarios are widely used in the literature, which makes the comparison with other research results easier. In this paper, four CMIP6 models were used to create an ensemble model, which represents the daily median values across the models. More information about the models can be found in Table 1.

**Table 1:** Climate models used to create the ensemble model

Model	Institution	More information
CanESM5	Canadian Centre for Climate Modelling and Analysis	Swart et al. 2019
GFDL-ESM4	Geophysical Fluid Dynamics Laboratory (GFDL)	Horowitz et al. (2018)
MPI-ESM1-2-LR	Max-Planck Institut für Meteorologie	Wieners et al. 2019
MRI-ESM2-0	Japan Meteorological Research Institute	Yukimoto et al. 2019

We evaluate the impacts of climate change on livestock, using historical simulations (from 1991 to 2010) and climate projections (scenario SSP5-8.5, from 2021 to 2080). The baseline period was chosen to consider the most recent climatology (20-years) and the availability of data from the climate models. The scenario SSP5-8.5 is considered as a pessimist scenario, with a higher increase of temperature by the end of the century, compared to other scenarios.

We divided the future projections into short- (2021–2040), medium- (2041–2060) and long-term (2061–2080). The variables used in this methodology are the daily temperature (tas) and the near-surface relative humidity (hurs). This dataset will be used to calculate the THI, as described in Section 2.3.

### Study area: Brazil

According to FAO (2023), livestock occupy about 26% of the global ice-free land with one-third of the cropland being used for feed production. In Brazil, livestock assumes an important position in the economy of the country. In 2021, crop and livestock production accounted for eight percent of Brazil’s Gross Domestic Product (GDP) (USDA, 2022). According to the USDA (2022), the value of Brazil’s agriculture, including cultivation of crops and livestock production, grew an average of eight percent annually over the past two decades (2000–2020), with agricultural output doubling and livestock production increasing threefold.

**Fig. 1:**
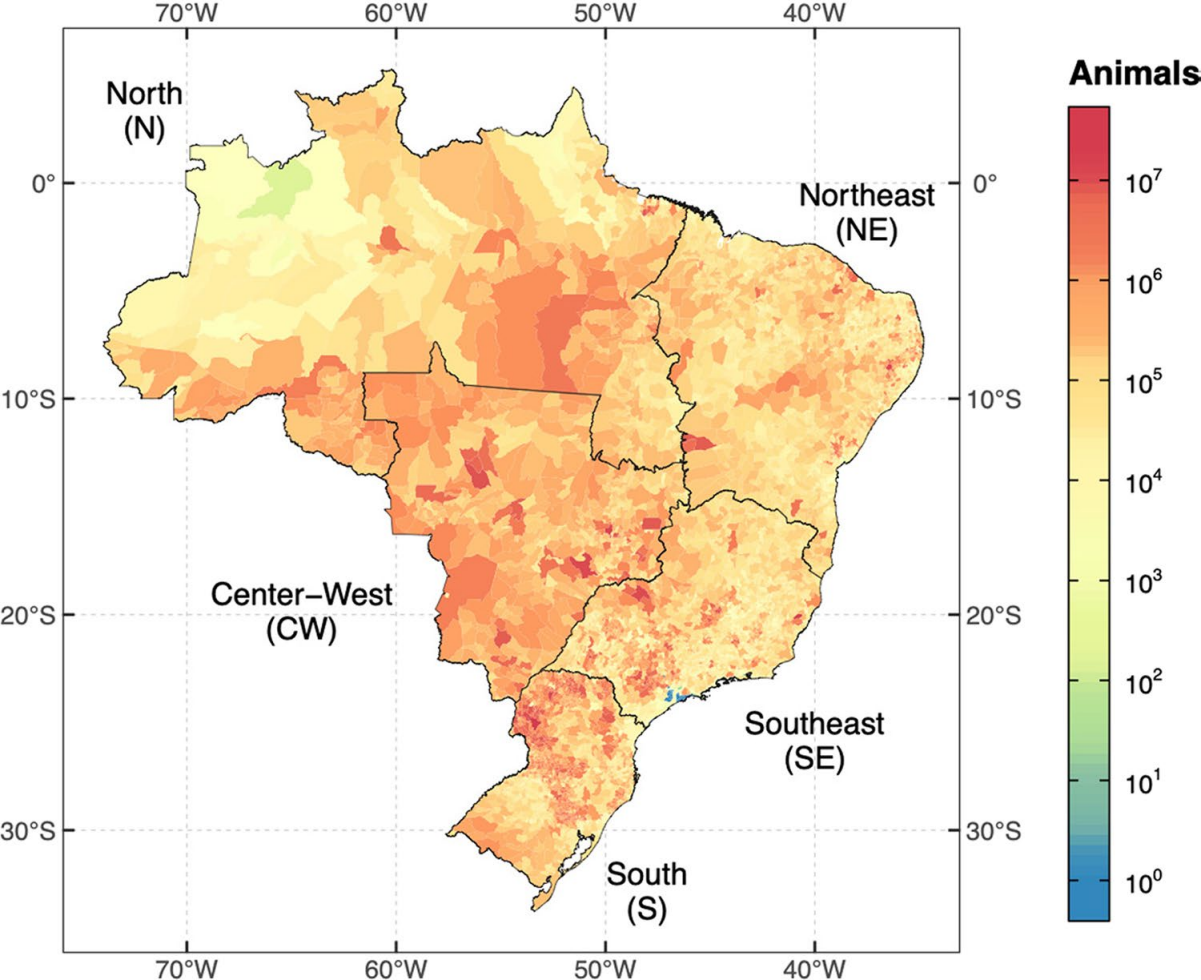
Number of livestock animals (cattle, buffaloes, horses, pigs, goats, sheep, chickens, quails) in 2022 by municipalities in the geographical regions of Brazil: North (N), Northeast (NE), Central-West (CW), Southeast (SE) and South (S). Source: PPM-IBGE (2022)

Brazil is divided in five geographical regions: North, South, Southeast, Center West, and Northeast, as indicated in Fig. 1. Due to the country's large territorial extension, animal production and the thermal environment differ between regions.

Brazil stands out among the largest producers and exporters of protein of animal origin, with emphasis on the production of cattle dairy, cattle beef, goats, sheep, pigs, poultry general, the focus of this research. Milk production in the country, estimated at 35.30 billion liters in 2021 (IBGE 2022), is distributed throughout almost the entire country. The Southeast, Central-West and South regions with the greatest production, mainly the states of Minas Gerais, Goiás, Paraná, Santa Catarina and Rio Grande do Sul (EMBRAPA 2023).

According to data from IBGE (2022), in the first quarter of 2022, 1.55 billion heads of broiler chickens were slaughtered. In this period, the South Region accounted for 60.2% of the national chicken slaughter, followed by the Southeast (19.2%), Central-West (14.7%), Northeast (4.3%) and North (1.6%). The production of chicken eggs was 977.20 million dozen, with the southeast and south regions standing out as the largest producers. For pig production, 13.64 million heads of pigs were slaughtered, with the South Region accounting for 66.0% of the national pig slaughter, in the 1st quarter of 2022, followed by the Southeast (18.8%), Central-West (13.9%), Northeast (1.2%) and North (0.1%).

Brazil is the largest beef exporter in the world and has a cattle herd of 234.3 million heads. Beef cattle production in the country is predominantly based on pastures. In the 1st quarter of 2022, 6.96 million heads of beef cattle were slaughtered. The Central-West Region presented the highest proportion of cattle slaughter in the period, 37.1% of the total, followed by the North (21.7%), Southeast (21.3%), South (11.4%) and Northeast (8.5%) (IBGE 2022).

According to EMBRAPA (2024), Brazil is the 18th largest producer of sheep and the 21st largest producer of goats in the world. Sheep and goat farming are significant activities, especially in the Northeast and South regions of Brazil. The South region produces around 90% of the goat herds and 60% of the sheep herds (EMBRAPA, 2024). In 2022, the herd population in the country was more than 12 million goats and more than 21 million sheep (IBGE 2022).

### Temperature humidity index (THI)

The thermal environment is one of the major climatic factors that affects animal production, and can be reproduced as a combination of air temperature, humidity, and air movement (Ames 1980). There is a thermal zone, where the animals exhibit optimum performance and minimal energy expenditure (Nardone et al. 2006). When the animal is suffering from an individual source of stress, the phenotypic response is called acclimation (Nardone et al. 2010).

Considering the impacts of climate change, it is notable that the animals across the world are outside this thermal zone. This means that extra energy will be required to maintain thermoregulation and production processes may become less effective (Joy et al. 2020; Godde et al. 2021). Research shows that milk production tends to be constant when the ambient temperature is within the thermoneutral zone, but drops linearly as the THI increases (Hempel et al. 2019; Lobeck et al. 2012). Therefore, when an animal is exposed to a heat stress they are not able to dissipate sufficient heat to keep homeothermy, leading to an increasing in respiration, pulse, heart rate, and body temperatures (Fregly, 2011; Nardone et al. 2010; Kadzere et al. 2002). This can lead to a reduction in the feed intake, reproduction efficiency, as well as changes in mortality and immune system function (Das et al. 2016; Sejian et al. 2018). This may become an additional challenge to a world that is already concerned with future food security under scenarios of climate change. Cheng et al. (2022) produced a literature review highlighting the Climate Change and Livestock Production. According to the authors, adaptation measures are essential to sustain the growing demand for livestock products, however their relevance depends on climatic conditions, the management of local production, as well as ensuring comfort and welfare conditions for the animals. At the same time, mitigation is key to limiting the future worsening of climate change and there are a number of possible strategies.

The environmental conditions that induce heat stress can be calculated using the temperature humidity index (THI), which is determined with a combination of ambient temperature and relative humidity (NRC 1971). The THI can be defined as NRC (1971).

1 $$THI=\left(1.8*T+32\right)-[\left(0.55-0.0055*RH\right)*\left(1.8+T-26\right)]$$

where T is the air temperature (°C), RH is the relative humidity (%) and THI is the Temperature humidity index.

The THI was applied in several researches across the world (Andrade et al. 2022b; Kang et al. 2020; Lallo et al. 2018). THI varies according to the animal species, as each animal species has different mechanisms to cope with high air temperature and relative humidity. Table 2 presents a compilation of thresholds for THI, classified as *Moderate*, *High* and *Extreme* heat stress (adapted from Thornton et al. 2021), considering relevant domesticated animal species in the Brazilian livestock context.

**Table 2:** THI onset of the stress level for different species (cattle dairy, cattle beef, goats, sheep, pigs, poultry-geral), classified as Moderate, High and Extreme heat stress

Species	Onset of the stress level			References
	Moderate	High	Extreme	
General1	72	78	90	Fuquay (1981)
Cattle-dairy	72	79	89	Dunn et al. (2014); Dash et al. (2016); Ranjitkar et al. (2020); Rahimi et al. (2021)
Cattle-beef	72	82	94	Mader et al. (2006); Valente et al. (2015)
Goats	70	79	89	Serradilla et al. (2018)
Sheep	72	78	90	McManus et al. (2016); Belhadj Slimen et al. (2019)
Pigs	75	79	84	Xin & Harmon (1998); Lallo et al. (2018); Mutua et al. (2020)
Poultry-general	73	81	85	Moraes et al. (2008)2

1General—THI value considered by the literature for all animals. 2THI limit table adapted from Thornton et al. (2021). Moraes et al. (2008) used five different categories for poultry-light and moderate discomfort were merged here.

To evaluate the impact of climate change in the livestock in Brazil, we used the THI onset of stress levels presented in Table 2 and calculated the number of days with moderate, high and severe stress for different animals in historical simulations and future projections from CMIP6. Therefore, we filtered the number of days per year with THI lower than 72 (e.g. moderate stress for general class), THI between 72 and 78 (high stress), and higher than 90 (extreme stress). From these values we constructed a climatology of the number of days with moderate, high and extreme stress, for each time-slice. We focused on days with extreme and high stress, and how they change according to each species and considering different time-slices.

## Results and discussions

### Climate change projections

**Fig. 2:**
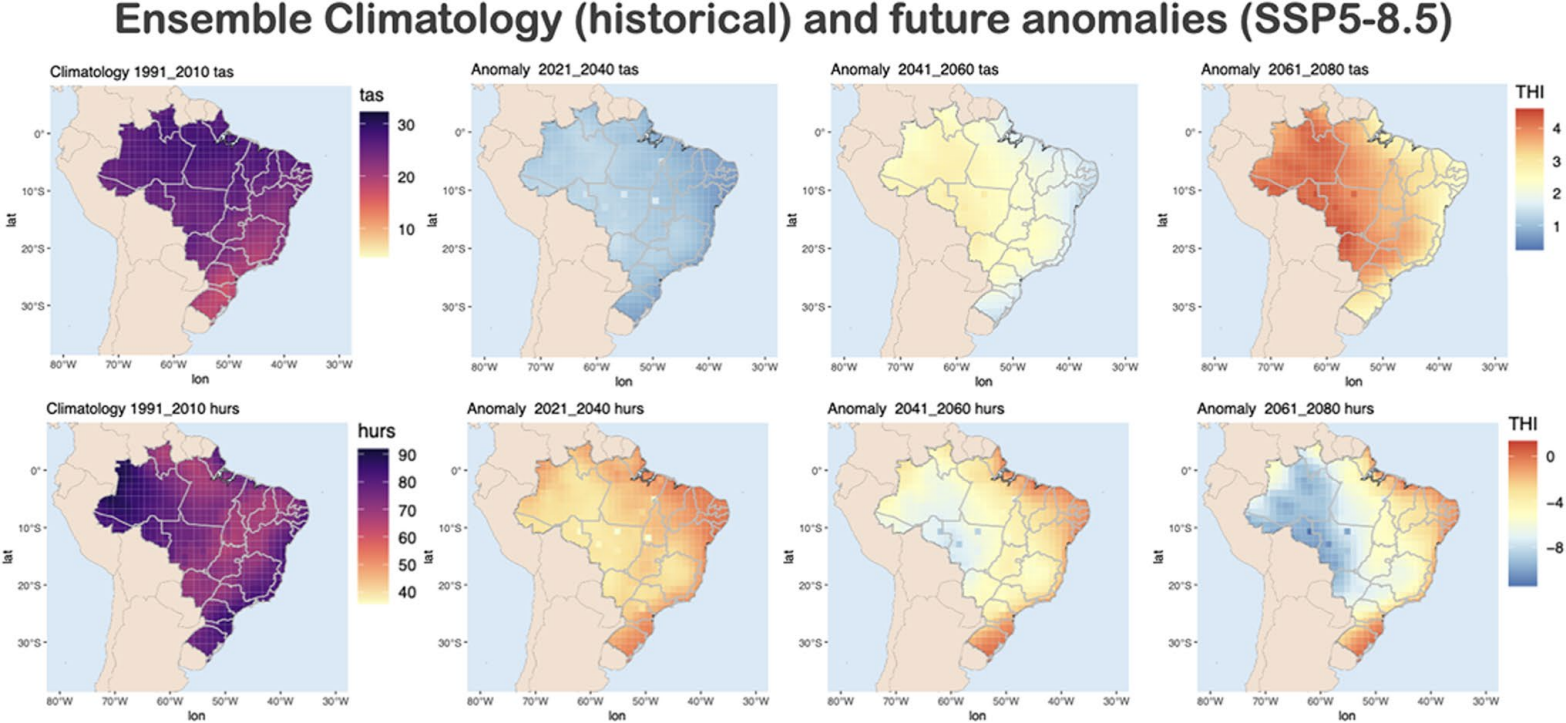
Daily mean temperature (tas, °C) and relative humidity (hurs, %) climatology for the baseline period (1991–2010) and anomalies between future and baseline period, considering short- (2021–2040), medium- (2041–2060) and long-term (2061–2080) simulations for SSP5-8.5

Figure 2 shows the climatology of temperature and relative humidity for the ensemble model (left) and the anomalies between the future projections (SSP5-8.5) and the historical period (right). As previously mentioned, the historical period included data from 1991 to 2010, and the future projections are divided into short- (2021–2040), medium- (2041–2060), and long-term (2061–2080). The anomalies are calculated based on the difference between future projection and historical period.

According to the climatology of temperature and relative humidity, we can notice that there is a great spatial variability of these variables across Brazil. For temperature, the lowest temperatures are found in South of Brazil, while for relative humidity the lowest relative humidity are found in central regions in Brazil.

In terms of anomalies, we can expect an increase in temperature in all projections, especially towards the end of the century. In the short-term, we expect an increase of 1 °C across the country. In the long-term however, we find more variability of this increase across the country, with highest values in the North and Central West part of Brazil, reaching values up to 4 °C. The increasing of temperature can be problematic to livestock, especially in the production phase, as it will require adaptation measures to provide comfort for the animals. We highlight that the values in Fig. 2 represent the average for the 20-years period evaluated. In terms of extreme events, the increase of temperature can be even higher, which will also have an impact on the livestock.

For animals kept outdoors, for example in pastures, an adaptation method with an adequate cost–benefit ratio is the provision of shade to reduce exposure to solar radiation and reduce thermal stress (Cheng et al. 2022). Sprinklers and foggers can also help reduce heat stress and are more effective in drier climates. Another example is the interaction of different methods, for example, the combination of sprinkling and a covered pen without an outdoor yard leads to a higher daily gain for hogs than sprinkling alone (Huynh 2005). For animals kept indoors, physical modification options may involve the use or addition of (1) ventilation systems, (2) heat-reducing construction materials (e.g., insulation), (3) orientation, and (3) forced air velocity associated with evaporative cooling (for example, misting, spraying and pad cooling). However, the cooling system has the best performance in terms of reducing thermal stress in hot and dry environments.

In terms of relative humidity, we mainly expect decreasing of this variable in the future, being this decreasing more pronounced towards the end of the century. The North and Central West regions are the regions where we expect the highest differences, with a decreasing of relative humidity of around 8%. For the Central West region this adds an additional challenge in the livestock production, as they already face problems with low relative humidity in the region, as the region tends to become drier and hotter (Hoffman, 2021).

### Climatology of THI and future projections

**Fig. 3:**
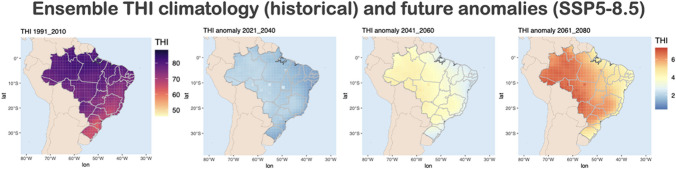
Daily mean THI (-) climatology for the baseline period (1991–2010) and anomalies between future and baseline period, considering short- (2021–2040), medium- (2041–2060) and long-term (2061–2080) simulations for SSP5-8.5

To assess the impact of climate change on heat stress, the THI was calculated for historical and future projections. Figure 3 shows the THI climatology for historical period, and anomalies between future projections and historical.

From Fig. 3, we can identify that the historical simulations show that THI is higher in the North, Northeast and Central West of Brazil. Considering the anomalies between future projections and historical simulations, we identify that in the time-slice 2021–2040, the THI may have an increasing of 2 [-]. For the time-slice from 2061–2080, we estimate higher increase of THI (up to 6 [-]) and higher spatial variability of index, compared to historical simulations. The results are aligned with results presented in Fig. 2, which also indicates that these regions are getting hotter and drier in the future.

**Fig. 4:**
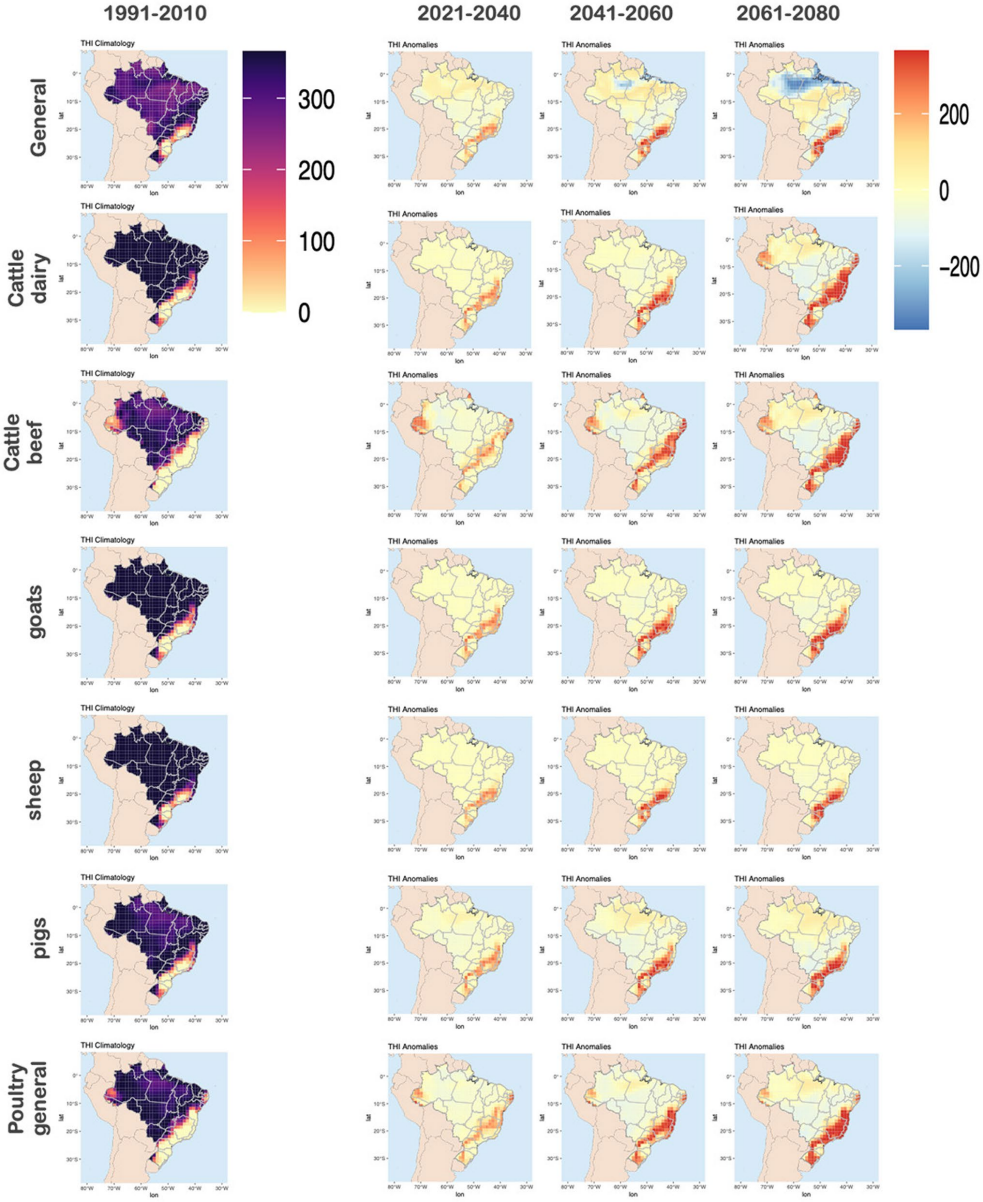
Number of days with high heat stress for each species (Historical, 1991–2010) and anomalies between future and historical for the short- (2021–2040), medium- (2041–2060), and long-term (2061–2080)

**Fig. 5:**
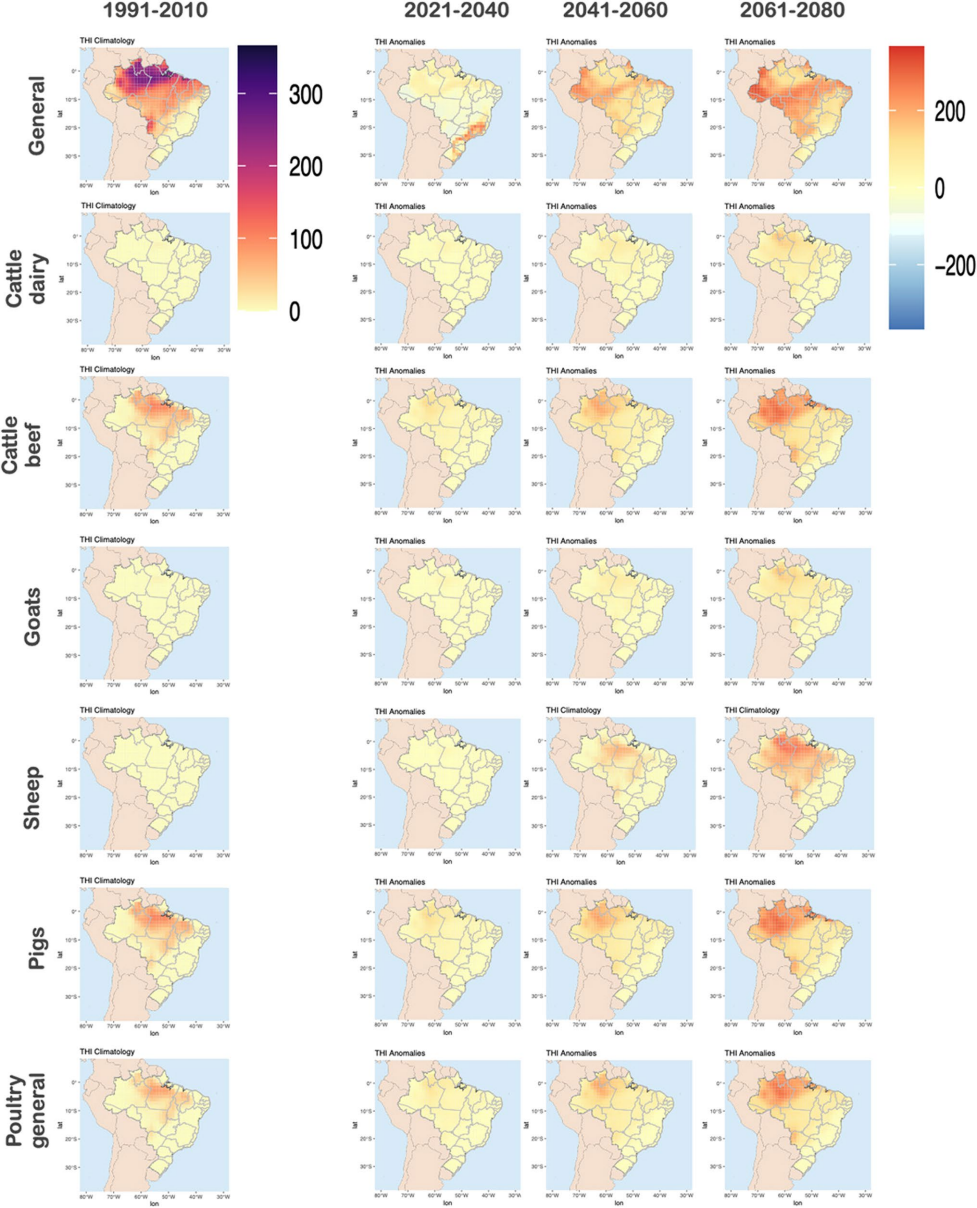
Number of days with extreme heat stress for each species (Historical, 1991–2010) and anomalies between future and historical for the short- (2021–2040), medium- (2041–2060), and long-term (2061–2080)

As defined in the methodology section, the risk can be divided into moderate, high and extreme stress. As identified in Fig. 3, the THI tends to increase in future scenarios. Therefore, the analysis of the number of days with moderate stress tends to decrease in the future for all evaluated species (not shown). Considering that the high and extreme stress are the classes with increasing THI trends and which are more harmful to the animals, the results focused on these classes. Figure 4 and Fig. 5 shows the number of days (average for 20-years) with high and extreme THI (respectively) for historical projections (left), and the anomalies for short-, medium-, and long-term, for different species (right).

In Fig. 4 we can observe that the highest occurrence of *high heat stress* are found in North, Northeast and Central West of Brazil, considering all the evaluated species. For all species, the number of days with *high heat stress* increases as we move towards the end of the century. The 2061–2080 time-slice indicates the worst case scenario, where most affected regions are South and Southeast, with an increase in the number of days per year with *high heat stress* higher than 200 days (for all considered species). For some species (e.g. *poultry general*, *cattle beef* and *cattle dairy*), there will be also an increase in the days with *high heat stress* in coastal areas of Northeast. The *general* results show a decrease of days with *high heat stress* in North (2061–2080 time slice), which is not seem for other specific species. This shows the importance of looking at different onset, more specific for the considered species.

In Fig. 5, we can observe that the highest occurrence of *extreme heat stress* are found in North of Brazil (*general*, *cattle beef*, *pigs* and *poultry general*). Similarly as presented in Fig. 4, the number of days with *extreme heat stress* increases as we move towards the end of the century. The 2061–2080 time-slice indicates the worst case scenario, where most affected region is North, with an increase in the number of days per year with *extreme heat stress* higher than 200 days.

**Fig. 6:**
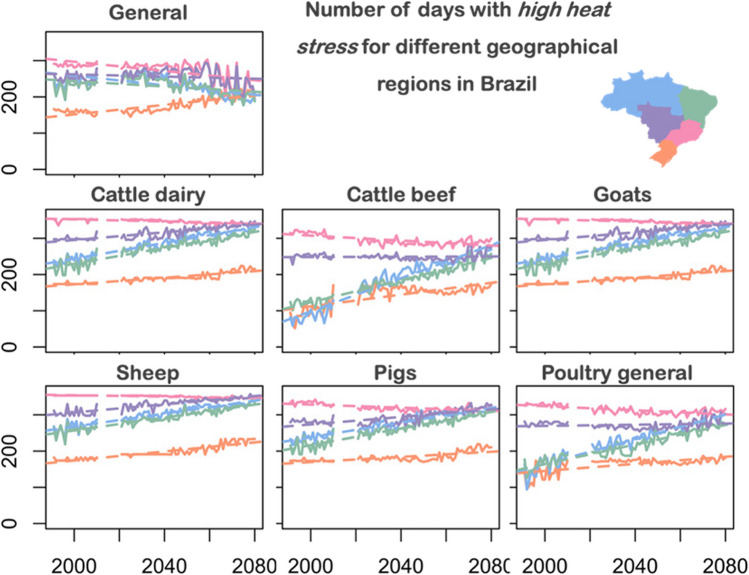
Number of days with high heat stress for different geographical regions in Brazil. Line colors correspond to the regions with the same color in the brazilian map (upper-right)

**Fig. 7:**
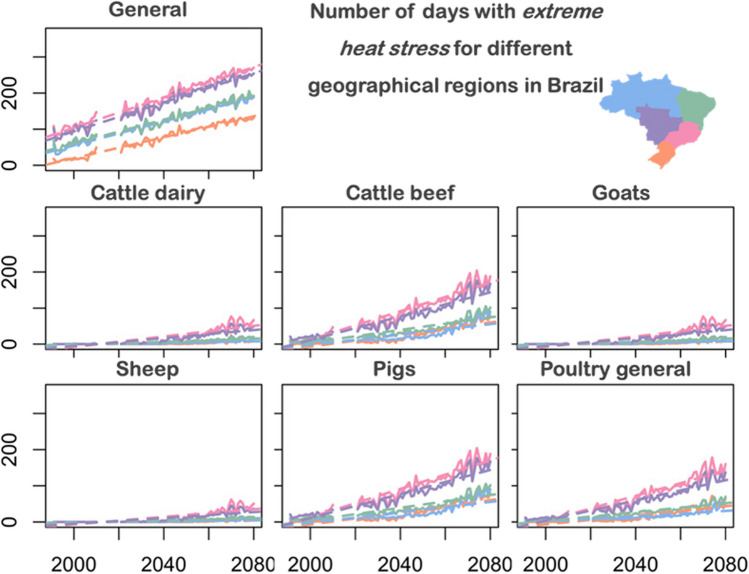
Number of days with extreme heat stress for different geographical regions in Brazil. Line colors correspond to the regions with the same color in the brazilian map (upper-right)

Figure 6 and Fig. 7 shows the number of days with *high (extreme) heat stress* for different geographical regions in Brazil. According to Fig. 6, the North and Northeast show an increase of *high heat stress* for *cattle dairy*, *cattle beef*, *goats*, *sheep*, *pigs* and *poultry in general*. However, for the species (*general)*, we identified decreasing trends for these regions. This can be explained in Fig. 7, where the *extreme heat stress* is increased in these regions for the *general category*. For the South, Southeast and Central West regions no relevant trends of *high heat stress* are identified for *cattle dairy*, *cattle beef*, *goats*, *sheep*, *pigs* and *poultry general*.

In Fig. 7 we identify increasing trends of *extreme heat stress* for all regions. However, the magnitude is different according to the region and the species evaluated. The regions with higher number of days with *extreme heat stress* are Southeast and Central West. These numbers are especially high for the species: *general*, *cattle beef*, *pigs*, *poultry general*. More than a third of the beef cattle herd is raised in the Central-West region of Brazil (PAM-IBGE 2019). According to a study carried out in Brazil by Zilli et al. (2020), the impacts of climate change affect the livestock sector through productivity losses and, to a lesser extent, through losses in the production of soybeans and corn used as livestock feed. This indicates the need for greater strategies on the part of rural producers to maintain better solutions for construction materials, shading, ventilation and cooling systems to ensure greater comfort and welfare for the animals.

**Fig. 8:**
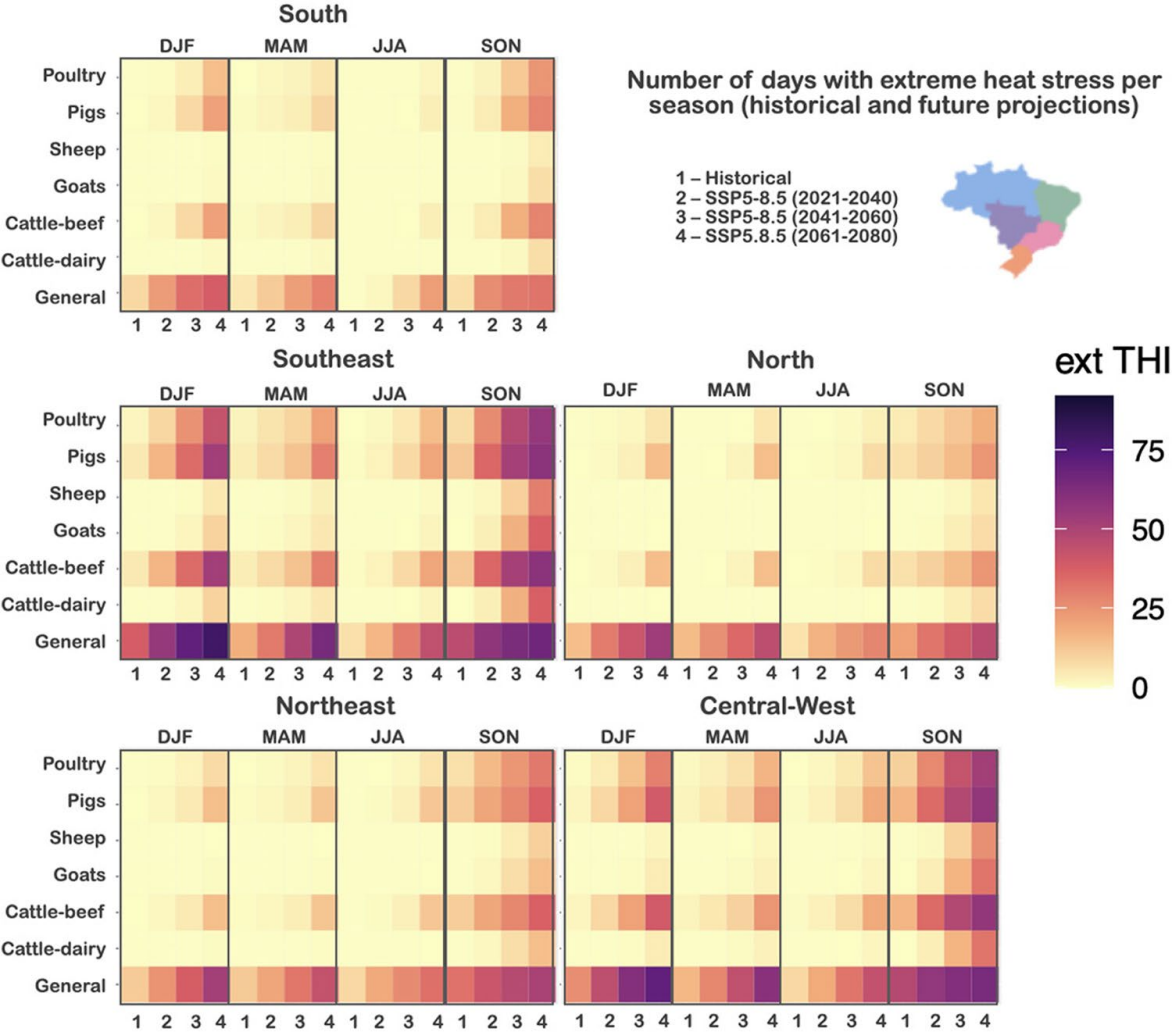
Number of days with extreme heat stress (ext THI) per season (DJF, MAM, JJA, and SON) for each specie, considering the historical simulation (1) and the projections for the SSP5-8.5 scenario for (2) short- (2021–2040), (3) medium- (2041–2060), and (4) long-term (2061–2080)

To evaluate the effect of climate change on the seasonality of the *extreme heat stress*, we also evaluate the different seasons for each region (Fig. 8). The heatmap presented in Fig. 8 shows the number of days of *extreme heat stress* for each specie (named in the scale as ext THI), considering the historical simulation and the projections for the SSP5-8.5 scenario for short- (2021–2040), medium- (2041–2060), and long-term (2061–2080). The seasons were defined as DJF (summer), MAM (autumn), JJA (winter) and SON (spring).

Figure 8 shows that the season with higher *extreme heat stress* is spring (SON), followed by summer (DJF). Southeast and Central-West will be the most affected areas, according to future projections. *Poultry*, *pigs*, *cattle-beef* and *general* are the species with higher impact due to climate changes.

## Conclusions

The goal of this paper is to evaluate the impacts of climate change by using the THI, which is widely used in research for regions with tropical and temperate climates. Based on the temperature and relative humidity of the air, the calculated THI values ​​reflect exposure to recorded heat levels. We evaluated the THI projections by using CMIP6 ensemble models for historical period, and short-, medium- and long-term projections in a pessimist scenario of climate change (SSP5-8.5). It is important to remember that this scenario, and therefore our results, can be seen as the worst case. Still, our results can help livestock producers to better prepare for impacts of climate change on the production.

The results presented in this paper show an increase of high heat stress in South and Southeast, and an increase of extreme heat stress in the North and Central-West areas of Brazil. The increase in extreme heat stress tends to occur mostly during spring and summer. This increase tends to vary considering the different evaluated species. Within the evaluated animal species, the species that seem to be more affected by climate changes are *Poultry*, *pigs*, *cattle-beef* and *general.* The differences between the results for the five geographic regions in Brazil suggests that different mitigation measures need to be considered to cope with future heat stress in livestock.

Stressful environments impair agricultural production, that is, animal growth, production and quality of milk and meat, egg production, weight, reproductive quality and performance, and metabolic and health status. In this context, our results suggest that it is strategically efficient to use measures to deal with environmental thermal stress. With climate changes indicating drier and hotter future conditions in Brazil, the heat stress in livestock may become an additional challenge to a world that is already concerned with future food security under scenarios of climate change. This situation may be even more problematic if the global society does not reduce meat consumption.

Regional changes in production in Brazil, observed in all regions and species considered, raise concerns regarding the availability of infrastructure and resources to accommodate them. The aspect to consider is that, due to climate changes, there will be an even greater need for cooling systems, with attention to rising water and electricity costs. More intense insertion of mechanized systems powered by renewable energy sources is also likely to reduce costs and potential increases in greenhouse gas emissions that would otherwise result in the use of fossil fuels. At the same time, food production in Brazil, the world's largest exporter of beef and soy, has been responsible for a large part of the country's greenhouse gas emissions. Most emissions are directly related to deforestation to convert native vegetation into pastures, being the main source of carbon released by Brazil into the atmosphere. Pollution from beef packing plants is also quite significant in the country. Measures related to food safety, animal welfare practices, societal acceptability and greenhouse effect reduction measures are essential for the food production chain as a whole.

As a limitation of this work, we highlight that we only considered scenario SSP5-8.5 and additional scenarios may provide useful insights into the range of possibilities. Additionally, the investigation of similar indexes that evaluate animal welfare, and local studies that include observational datasets and high-resolution projections could potentially decrease the uncertainties associated with the projections.
